# ECFuse: Edge-Consistent and Correlation-Driven Fusion Framework for Infrared and Visible Image Fusion

**DOI:** 10.3390/s23198071

**Published:** 2023-09-25

**Authors:** Hanrui Chen, Lei Deng, Lianqing Zhu, Mingli Dong

**Affiliations:** 1 Key Laboratory of the Ministry of Education for Optoelectronic Measurement Technology and Instrument, Beijing Information Science & Technology University, Beijing 100192, China; hanruichen@bistu.edu.cn (H.C.); dongml@bistu.edu.cn (M.D.); 2 Beijing Laboratory of Optical Fiber Sensing and System, Beijing Information Science & Technology University, Beijing 100016, China; 3 Guangzhou Nansha Intelligent Photonic Sensing Research Institute, Guangzhou 511462, China

**Keywords:** infrared–visible image, image fusion, nonsubsampled shearlet transform, deep learning

## Abstract

Infrared and visible image fusion (IVIF) aims to render fused images that maintain the merits of both modalities. To tackle the challenge in fusing cross-modality information and avoiding texture loss in IVIF, we propose a novel edge-consistent and correlation-driven fusion framework (ECFuse). This framework leverages our proposed edge-consistency fusion module to maintain rich and coherent edges and textures, simultaneously introducing a correlation-driven deep learning network to fuse the cross-modality global features and modality-specific local features. Firstly, the framework employs a multi-scale transformation (MST) to decompose the source images into base and detail layers. Then, the edge-consistent fusion module fuses detail layers while maintaining the coherence of edges through consistency verification. A correlation-driven fusion network is proposed to fuse the base layers containing both modalities’ main features in the transformation domain. Finally, the final fused spatial image is reconstructed by inverse MST. We conducted experiments to compare our ECFuse with both conventional and deep leaning approaches on TNO, LLVIP and $M^{3}\mathrm{FD}$ datasets. The qualitative and quantitative evaluation results demonstrate the effectiveness of our framework. We also show that ECFuse can boost the performance in downstream infrared–visible object detection in a unified benchmark.

## 1. Introduction

Image fusion is a basic and popular topic in image processing that seeks to generate informative fused images by integrating essential information from multiple source images. Infrared and visible image fusion (IVIF) is one of the important sub-categories of image fusion [1]. IVIF focuses on preserving detailed texture and thermal information in the input images [2]. The fused images can mitigate the disadvantages of visible images, being susceptible to illumination and other environmental conditions, as well as avoiding the issue of infrared images lacking texture.

Numerous methods have been proposed to tackle the challenge of IVIF [3,4,5,6,7,8,9,10,11]. These methods can be mainly categorized into deep learning-based approaches and conventional methods. Deep learning methods are becoming increasingly popular in the fusion task due to their ability to extract high-level semantic features [5,7,10,12], but there is still a need for improvement in preserving complex and irregular edges within images. Infrared and visible images, coming from the same scene, inherently share statistical co-occurrent information, such as background and large-scale features. Transformer-based deep learning frameworks are good at extracting global features from inputs, so they are well suited for fusing the main features of infrared and visible images.

Conventional methods offer better interpretability, and their rich prior knowledge enables the design of fusion techniques that effectively preserve high- and low-frequency information. But they may suffer from high design complexity. Conventional fusion methods can be generally divided into several categories according to their adopted theories [13], i.e., multi-scale transformation (MST), saliency-based methods, sparse representation, subspace, etc. One of the most active and well-established fields for image fusion is MST. It decomposes input images into a base layer containing low-frequency main feature and detail layers containing high-frequency texture and edges. Some studies demonstrated that MST-based methods are aligned with human visual characteristics [14,15] and this property enables fused images to have an appropriate visual effect. Regarding MST-based fusion schemes, many methods employ weighted averaging or maximum value schemes. Simple weighted averaging may diminish the contrast of salient regions, while the pixel-wise application of the maximum value strategy may not adequately preserve the continuity of edges and textures.

To this end, we propose a novel edge-consistent and correlation-driven fusion framework. Specifically, we first employ a MST-based method to decompose the input images into low-frequency base layers and high-frequency detail layers. Subsequently, the edge-consistent fusion module is proposed to adaptively fuse the detail layers that mainly contain edges and textures. For the base layers, which are more suitable for extracting main features employing deep learning, a correlation-driven deep learning network trained on MST domain images is proposed to extract the base layers feature and fuse them. Finally, the inverse MST is employed to reconstruct the final fused image. By combining deep learning and conventional methods, their advantages can be kept. The effectiveness of the combination of deep learning and conventional methods within our proposed framework is validated through extensive experiments.

Our main contributions are summarized as follows:

1. We propose a novel edge-consistent and correlation-driven fusion framework. We leverage the advantages of conventional methods and deep learning methods. Experiments demonstrate our framework’s effectiveness and boost the performance in the downstream infrared–visible object detection task.
2. We propose a novel edge-consistent fusion method. The proposed method aims to transfer salient edges and textures from the source image to the fused image while keeping edges intact, rather than merely fusing the pixel values from each source image.
3. We propose a correlation-driven network that is applicable in the MST domain. The correlation loss function is introduced to enforce the global encoder in extracting shared cross-modal information, while the residual architecture enables the encoder to preserve distinctive features for each modality.

## 2. Related Works

While it is common and feasible to perform object detection or segmentation tasks using either infrared or visible images individually [16,17], Tang’s research [18] demonstrates that the fusion of infrared and visible images can improve the performance in downstream tasks. This section briefly reviews the representative works of MST-based IVIF methods and deep learning-based IVIF approaches.

### 2.1. MST-Based Fusion Methods

MST contains many methods, such as wavelet transform, contour transform, nonsubsampled contourlet transform (NSCT), and nonsubsampled shearlet transform (NSST). Various MST-based methods have been applied to image fusion [14,19]. NSCT was proposed by Da Cunha et al. [20], and is based on contourlet transform [21]. NSCT has been widely applied in infrared and visible image fusion. The entropy of the square of the coefficients and the sum of the modified Laplacian were utilized in the frequency domain [19]. Easley et al. proposed NSST [22], which is realized by nonsubsampled Laplacian pyramid and shearing filters. Zhang et al. [9] proposed a new image fusion method regarding global–regional–local rules applied to overcome the problem of wrongly interpreting the source image. The source images are statistically correlated by the G-CHMM model, R-CHMM model, and L-CHMM model in the high subband region. High-pass subbands were fused by global–regional–local CHMM design and choose-max rules based on the local gradient measure. Finally, the fused images were extracted by exploiting the inverse NSST. Liu X et al. [3] proposed a multi-modality medical image fusion algorithm that utilizes a moving frame-based decomposition framework (MFDF) and the NSST. The MFDF is applied to decompose the source images into texture components and approximation components. The maximum selection fusion rule is employed to fuse the texture components, aiming to transfer salient gradient information to the fused image. The approximate components are merged using NSST. Finally, a component synthesis process is adopted to produce the fused image. Liu et al. proposed an image fusion algorithm based on NSST and modified-spatial frequency (MSF) [4]. It selects the coefficients with greater MSF to combine images when high-frequency and low-frequency subbands of source images are compared. Miao et al. [23] proposed an image fusion algorithm based on the NSST. The algorithm employs an average fusion strategy for the low-frequency information fusion and a novel method to fuse high-frequency information.

Lots of MST-based fusion methods utilize weighted averaging or maximum value strategies. However, simple weighted averaging may reduce the contrast of salient regions, and the simple maximum value strategy is applied pixel-wise, which may not preserve the continuity of edges and textures. To tackle these limitations, we propose an edge-consistency fusion method. This method incorporates the activity rules to preserve the brightness of salient edges and achieves texture continuity and integrity through consistency verification.

### 2.2. Deep Learning-Based Fusion Methods

The convolutional neural network (CNN) is a commonly used deep learning network model. In STDFusionNet [2], a salient target mask is employed to enhance the contrast information from the infrared image in the fused image. This approach aims to achieve a significant injection of contrast information. SeAFusion [7] is a novel semantic-aware framework for fusing infrared and visible images, achieving outstanding performance in image fusion and advanced visual tasks. These methods leverage CNN to extract features and perform fusion operations, enabling the effective integration of information from different modalities or sources. FusionGAN [6] is a groundbreaking method that applies a generative adversarial network (GAN) to the field of image fusion. It establishes a generative adversarial framework between the fused image and visible image, allowing the fused image to acquire texture and structure in a more enhanced manner. Following FusionGAN, there have been numerous fusion methods inspired by GAN, such as TarDal [5]. Additionally, a wide range of fusion methods based on autoencoders (AE) have been proposed by researchers. These methods commonly employ AE to extract features from source images and achieve image reconstruction. AE can capture relevant information and reconstruct images effectively, making them a popular choice in fusion techniques. DenseFuse [8] uses the structural strength of DenseNet [24], resulting in an effective fusion outcome. DIDFuse [10] is also an AE-based image fusion method that replaces the transformers and inverse transformers with encoders and decoders.

The deep learning methods excel at extracting high-level semantic features, and the AE-based approaches are capable of capturing global information from images, making the extraction of shared features between infrared and visible images more effective, such as background and large-scale features. This advantage makes them well-suited for fusing the main features of images. Therefore, we design a correlation-driven AE-based method for fusing the main information of images.

## 3. Method

**Figure 1:**
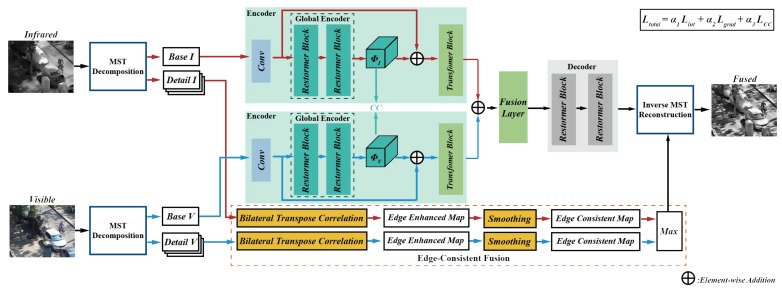
The schematic diagram of the proposed fusion framework.

Figure 1 depicts the schematic diagram of the proposed fusion framework. NSST is one of the most popular MST methods, so we choose it as an implement to validate the working of our method. Firstly, we employ NSST to decompose the source images into their respective base and detail layers. Subsequently, the detail layers of infrared and visible images are adaptively enhanced and fused using bilateral transpose consistency module. Then, the base layers are fused by a correlation-driven network. Finally, the fused base and detail layers are reconstructed into the final fused image using the corresponding inverse transform.

### 3.1. NSST Decomposition

**Figure 2:**
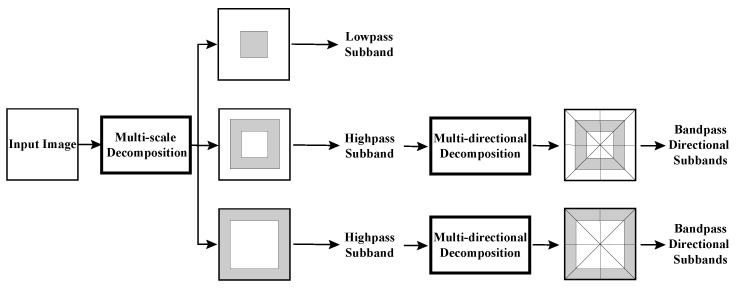
The schematic diagram of NSST decomposition.

As depicted in Figure 2, NSST decomposes the source image into a series of high- and low-frequency sub-images. It can be divided into two major steps: (1) Multi-scale decomposition can obtain subbands by using the non-subsampled (NS) pyramid filter. (2) Shearlet transform (ST) is employed to obtain multi-directional decomposition. The ST is close to the optimal sparse representation. The synthetic expansion of the affine system is described as follows:

$$\begin{array}{c} \Lambda_{AB}\left(\psi \right)=\{\psi_{j,l,k}\left(x\right)={\left|\det A\right|}^{j/2}\psi \left(B^{l}A^{j}x-k\right):j,l\in Z,k\in Z^{2}\}, \end{array}$$

where $\psi_{j,l,k}$ represents a composite wavelet, *A* denotes the anisotropy matrix for multi-scale decomposition, and *B* is a shear matrix for directional analysis. *j*, *l* and *k* are the scale, the direction of decomposition, and the shift parameter, respectively.

The layer corresponding to the lowest frequency is referred to as the base layer, while the layers corresponding to higher frequencies are referred to as detail layers.

### 3.2. Edge-Consistent Detail Layers Fusion

**Figure 3:**
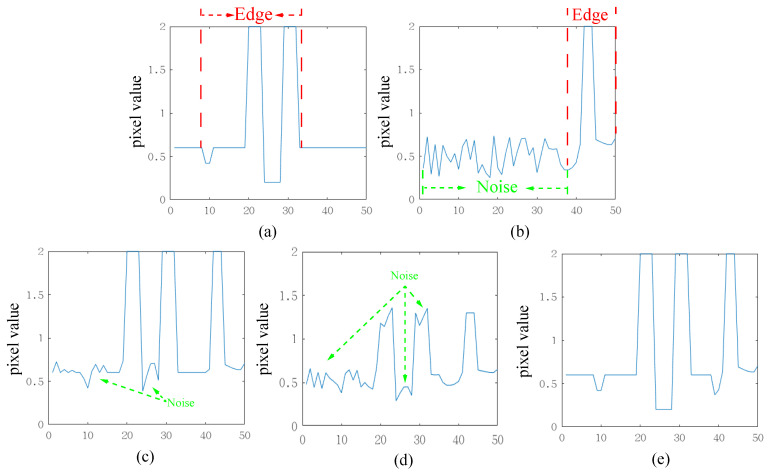
Schematic diagram of the fusion results of our proposed method. (**a**) Visible. (**b**) Infrared. (**c**) Max fusion scheme. (**d**) Average fusion scheme. (**e**) Our fusion scheme.

Existing simple average or maximum fusion schemes are performed on each pixel in the spatial image. However, detail layers obtained from NSST contain rich directional texture information, and using a simple scheme does not fully exploit this directional information. To provide a clear illustration of this effect, Figure 3 offers a one-dimensional signal example. The visible image predominantly encompasses regular edge signals, whereas the infrared image includes not only edge signals but also some noise. Employing simple maximum or average strategies would both result in fused outcomes containing noise. When the noise value exceeds the valuable signal in the other input image, the maximum strategy would be entirely submerged by noise, and the average strategy would diminish the value of the valuable edge information. In contrast, our proposed method retains edge information from the input images, even in the presence of noise interference. Our proposed edge-consistent fusion module consists of three main parts: bilateral transpose correlation, smoothing, and activity-based fusion. It is important to note that both bilateral transpose correlation and smoothing are designed to obtain the activity map, rather than serving as the images for subsequent fusion. The edge-consistent fusion module preserves the integrity of edges and textures while avoiding artifacts in the fusion result.

**Figure 4:**
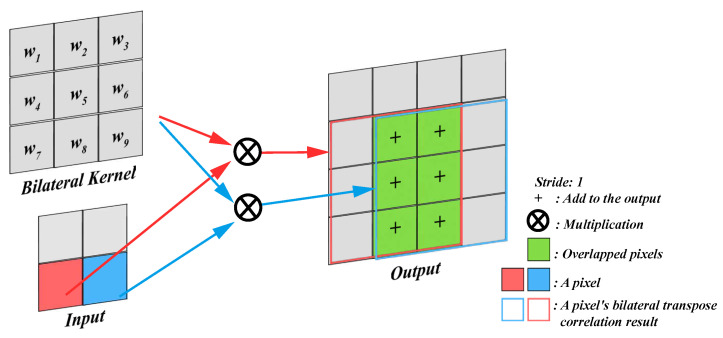
The schematic diagram of bilateral transpose correlation.

Firstly, the schematic diagram of the transpose correlation is shown in Figure 4. The larger absolute values in the detail layers correspond to sharper brightness changes and thus to the salient features in the image, such as edges, lines, and region boundaries. It can be observed that the overlapping regions during the kernel movement add up. When the pixels represented by red and blue belong to the edge, the accumulation will further enhance the edge. When both of these pixels do not belong to the edge, the accumulated value is relatively smaller. The size of the output image for transposed correlation can be calculated using Equation (1).

(1) O=(I−1)×s+k,

where *O* represents the size of the output image, *I* represents the input size, *k* represents the kernel size, and *s* represents the stride size. In this study, we simply choose *s* as 1 and *k* as 3.

The design of the kernel is crucial for the performance of the transpose correlation. Simple fixed kernels, while computationally efficient, often yield limited results. Another commonly used Gaussian filtering kernel considers only spatial distances, neglecting sharp changes in edges, leading to blurred edges. Bilateral filtering is a successful method, but its purpose is to filter noise rather than being specifically designed for image fusion. Therefore, we propose a novel non-iterative kernel construction method suitable for transpose correlation. This method considers both value distances and spatial distances, enabling the adaptive enhancement of edges and textures.

To obtain a bilateral kernel that can enhance both edges and textures, the value distance matrix is defined as in Equation (2). Since the pixel values of the edges themselves are larger or smaller, their corresponding pixel values should be less influenced by the surrounding non-edge pixels. Therefore, choosing the cube of the value distance is necessary to reduce the weight of the surrounding pixels. And if the surrounding pixels all belong to edges, they will mutually enhance each other’s effects:

(2) $$\begin{array}{c} G_{s}\left(x,y\right)=\exp\left(-\frac{|x-\mu_{x}{|}^{3}+{\left|y-\mu_{y}\right|}^{3}}{2\sigma_{s}^{2}}\right), \end{array}$$

where $G_{s}$ represents the value distance matrix, $\mu_{x}$ denotes the current window’s center x-coordinate and $\mu_{y}$ denotes the current window’s center y-coordinate. The parameter $\sigma_{s}$ represents the standard deviation of the spatial distance.

To consider the pixel value differences in the image, the value distance matrix is defined as given in Equation (3). As the variations in the edge regions are significant, it is essential to emphasize the extent of the pixel value changes, apart from considering the spatial distances. Taking the grayscale image as an example, the pixel value changes often exceed 100, which may lead to very small values in the numerator. To avoid an unnatural reduction in pixel values, normalization is performed. Inspired by softmax, we employ an exponential function for the normalization, which makes the value distance matrix more sensitive to pixel value changes:

(3) $$\begin{array}{c} G_{v}\left(x,y\right)=\frac{\exp\left(-\frac{{\left(I\left(x,y\right)-I\left(\mu_{x},\mu_{y}\right)\right)}^{2}}{2\sigma_{v}^{2}}\right)}{\sum_{(x,y)\in Q}\exp\left(-\frac{{\left(I\left(x,y\right)-I\left(\mu_{x},\mu_{y}\right)\right)}^{2}}{2\sigma_{v}^{2}}\right)}, \end{array}$$

where $G_{v}$ represents the value distance matrix, $I(\mu_{x},\mu_{y})$ denotes the pixel value corresponding to the current kernel center position, $\sigma_{v}$ represents the value distance standard deviation, and *Q* represents the window corresponding to the current kernel.

With the spatial distance matrix and value distance matrix of the same size now defined, the bilateral kernel can be formulated as shown in Equation (4). This kernel takes into account both the differences in the pixel values between different pixels and the influence of the spatial distances. The bilateral kernel is utilized in the bilateral transpose correlation:

(4) $$\begin{array}{c} K\left(x,y\right)=\frac{G_{s}\left(x,y\right)\times G_{v}\left(x,y\right)}{\sum_{(x,y)\in Q}\left(G_{s}\left(x,y\right)\times G_{v}\left(x,y\right)\right)}, \end{array}$$

where the *K* is the bilateral kernel.

After the bilateral transpose correlation processing, the output is larger than the size of the source image, so it needs to be restored to the same size as the source image. The enhanced edges through the bilateral transpose correlation exhibit more pronounced distinctions between the infrared and visible images. However, the accentuation of the edges can potentially introduce artifacts in the vicinity of the edges. So, smoothing is introduced to ensure smoother transitions between edges and surrounding pixels, preserving edges while achieving a more natural fusion outcome. This step accomplishes the consistency verification of edges and their neighboring regions. The values in the smoothing kernel are set to 1 in this paper. The smoothing is defined as Equation (5):

(5) $$\begin{array}{c} M=\sum_{i=0}^{k}\sum_{j=0}^{k}I\left(i,j\right)\times K\left(k-i,k-j\right), \end{array}$$

where *M* represents the result of the correlation, *I* denotes the input image, *K* refers to the correlation kernel, and *k* represents the size of the correlation kernel.

**Figure 5:**
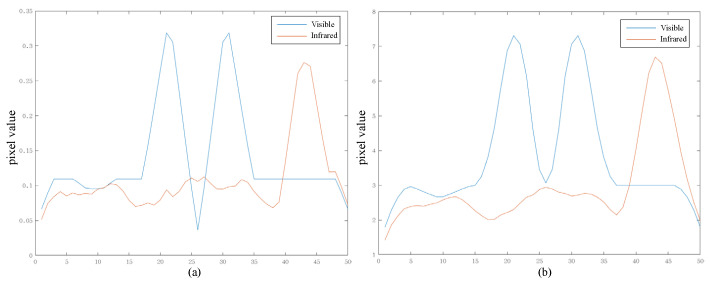
Schematic diagram of the results of edge-consistent module. (**a**) Bilateral transpose correlation result. (**b**) Smoothing result.

After processing the infrared and visible detail layers described above, we obtain corresponding images with enhanced edges and textures. In Figure 5, to visually demonstrate the effects of bilateral transpose correlation and smoothing, we provide an example using the one-dimensional signal from Figure 3. From Figure 5, it is evident that the bilateral transpose correlation amplifies the differences in the edges of the image. However, a little noise in the infrared image is still greater than the edges in the visible image. Then, smoothing raises all edges above the noise values, ensuring that the edges in the fusion result are continuous and intact.

To obtain the activity map, we define higher pixel values in the grayscale image as having higher activity and retain the positions with higher activity. The following formula is designed to generate the activity map:

(6) $$\begin{array}{c} M_{A}=\mathrm{sgn}\left(M_{v}-M_{i}\right), \end{array}$$

where $M_{A}$ is the activity map. $M_{v}$ and $M_{i}$ represent the enhanced visible detail layers and enhanced infrared detail layers, respectively.

The fused detail layer can be obtained by the following equation:

(7) $$\begin{array}{c} M_{F}\left(i,j\right)=\left\{\begin{array}{cc} M_{v}\left(i,j\right), & \mathrm{if}\;M_{A}\left(i,j\right)=1\\ M_{i}\left(i,j\right), & \mathrm{otherwise} \end{array}\right.. \end{array}$$

**Figure 6:**
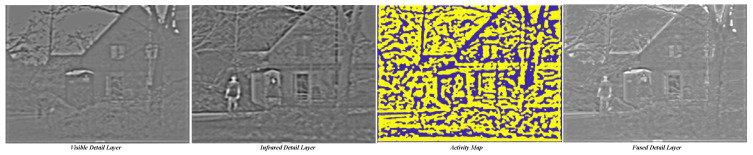
Visualization of the edge-consistency fusion result.

Figure 6 presents the visualized results of the aforementioned process. The NSST decomposition yields multiple detail layers, each corresponding to different scales and orientations of details. We illustrate the effectiveness of our proposed approach by selecting one of these detail layers. As observed in the figure, the source visible and infrared detail layers encompass distinct information. Following the application of the bilateral kernel, edge and texture information receive enhancement. The coherence in the chosen pixels, evident in the activity map, underscores the advantage of regionally consistent fusion, ensuring the continuity and integrity of edges and textures. The ultimate fused result adeptly not only retains the principal features of both infrared and visible images but also captures intricate and comprehensive details.

Note that edge-consistency fusion is a generic multi-modal image fusion method, and we only take the IVIF task as an example to explain its operation.

### 3.3. Correlation-Driven Base Layers Fusion

Both the infrared and visible images are captured from the same scene, thus containing statistically similar information such as background and objects. However, due to their different modalities, they also possess independent information, such as the rich textures in the visible images and the thermal radiation information in the infrared images. Therefore, our objective is to promote the extraction of modality-specific features and modality-shared features by constraining the correlation between different modality images.

The correlation-driven network contains three main modules, i.e., a transformer-based encoder for feature extraction, a fusion layer to fuse visible and infrared features, and a decoder for generating fusion images.

#### 3.3.1. Encoder

The residual architecture design of the encoder draws inspiration from ResNet [25], enabling the global encoder to extract shared cross-modal features, while also ensuring that the encoder captures modality-specific features for each modality.

First, we define some symbols for clarity in the formulation. The input paired infrared and visible images are denoted as $I\in R^{H\times W}$ and $V\in R^{H\times W\times 3}$. The global feature encoder and transformer block are represented by G(·) and P(·), respectively.

**Global encoder.** The global encoder aims to extract global features $\left\{\Phi_{V},\Phi_{I}\right\}$ from visible and infrared images {V,I}, i.e.,

(8) $$\begin{array}{c} \Phi_{V}=G\left(V\right),\Phi_{I}=G\left(I\right); \end{array}$$

The Restormer block can extract global features by applying self-attention across the feature dimension. So, we employ it to extract cross-modality global features without increasing too much computation. The detailed architecture of the Restormer block can be referred in Appendix A or the original paper [26].

**Transformer block.** The Transformer block receives the output of the residual structure network, retaining both the shared features across modalities and the distinct characteristics within different modalities. Additionally, the Transformer block employs the self-attention mechanism, enhancing the network’s ability to focus on the most relevant features for effective fusion:

(9) $$\begin{array}{c} \Phi_{VP}=P\left(\Phi_{V}\right), \end{array}$$

where $\Phi_{VP}$ is the encoded feature of *V*. And the infrared feature can be obtained similarly. Because the balance of performance and computational efficiency is important, the LT block [27] is chosen as the basic unit of the transformer block. The LT block shrinks the embedding to reduce the number of parameters while preserving the same performance.

#### 3.3.2. Feature Fusion Layer

The features of visible and infrared images are combined using an element-wise average strategy, which is then used as the input to the fusion layer. Considering that the inductive bias for feature fusion should be similar to feature extraction in the encoder, we also employ LT blocks for the fusion layer:

(10) $$\begin{array}{c} \Phi_{F}=F\left(\Phi_{VP}⊕\Phi_{IP}\right), \end{array}$$

where F represents the fusion layer. ⊕ is the element-wise addition.

#### 3.3.3. Decoder

The decoder D(·) reconstructs the features into the fused base layer:

(11) $$\begin{array}{c} F=D(\Phi_{F}). \end{array}$$

Since the inputs here involving cross-modality features, we keep the decoder structure consistent with the design of the global encoder.

#### 3.3.4. Loss Function

There is no ground truth for the IVIF task. We introduce the intensity loss $L_{int}$, gradient loss $L_{grad}$, and correlation loss $L_{CC}$ to constrain the visual quality of the fusion results.

The full objective function of the progressive fusion network is a weighted combination of the intensity loss, gradient loss and correlation loss, which is expressed as follows:

(12) $$\begin{array}{c} L_{total}=\alpha_{1}L_{int}+\alpha_{2}L_{grad}+\alpha_{3}L_{CC}, \end{array}$$

where $\alpha_{1}$, $\alpha_{2}$ and $\alpha_{3}$ are the tuning parameters.

The intensity loss quantifies the disparity between the fused images and the more salient regions within the infrared and visible images. Hence, we formulate the intensity loss as follows:

(13) $$\begin{array}{c} L_{int}=\frac{1}{HW}{∥F-\max\left(I,V\right)∥}_{1}, \end{array}$$

where *H* and *W* are the height and width of the input image, respectively. ${∥\cdot ∥}_{1}$ stands for the $l_{1}$-norm. max(·) denotes the element-wise maximum selection.

Moreover, we expect the fused image to maintain the optimal intensity distribution and preserve abundant texture details. The optimal texture of the fused image can be expressed as a maximum aggregate of the infrared and visible image textures. Therefore, a texture loss is introduced to force the fused image to contain more texture information, which is defined as follows:

(14) $$\begin{array}{c} L_{grad}=\frac{1}{HW}∥|\nabla F|-{\max(|\nabla V|,|\nabla I|)∥}_{1}, \end{array}$$

where ∇ indicates the Sobel gradient operator.

The above losses aim to ensure that the fusion results closely resemble the source images. However, they do not explicitly utilize the prior knowledge that the two modalities correspond to the same scene. Given that both infrared and visible images capture the same scene, it is evident that the background and common large-scale features are correlated. To address this, we introduce a correlation loss, ensuring that the global encoder learns related information while also easing the subsequent modules’ task of extracting modality-specific features:

(15) $$\begin{array}{c} L_{CC}=\frac{1}{CC(\Phi_{V},\Phi_{I})+\mu }, \end{array}$$

where CC(·,·) is the correlation coefficient operator, and μ here is set to 2 to ensure that this term always remains positive.

In conclusion, our correlation-driven fusion network effectively preserves salient regions and details from the source images while actively focusing on the correlation between the two modalities. Therefore, this network utilizes the shared and distinct information from both modalities, leading to meaningful and efficient fusion results.

After the fusion of detail and base layers, the inverse NSST is employed to reconstruct the spatial final fused image.

## 4. Experimental Results and Analysis

We test the performance of our proposed framework on publicly available datasets and compare it with thirteen state-of-the-art or well-known methods, including seven deep learning methods, i.e., FusionGAN [6], DIVFusion [28], DenseFuse [8], DIDFuse [10], STDFusionNet [2], SeAFusion [7], TarDal [5], and six conventional methods, i.e., Wavelet [29], ADF [30], TIF [31], LatLRR [32], FPDE [33], IFEVIP [34]. All methods were implemented using publicly available codes, with parameters set according to the original paper. The experiments were conducted on a platform equipped with an Intel(R) Core(TM) i9-10980XE CPU @ 3.00 GHz and a NVIDIA GeForce RTX 3090 GPU.

### 4.1. Datasets and Settings

The TNO [35] dataset is utilized for testing our framework. We randomly select 42 pairs of typical images from it. This dataset contains multispectral nighttime images of various military-related scenes, which are registered using different multiband camera systems. With the development of technology, the image resolution is constantly improving, so we also test with the newer LLVIP [36] dataset. This dataset is a registered infrared–visible image dataset, with most image pairs taken under low light conditions. The dataset consists of 3464 registered pairs of infrared–visible image pairs. For the training of the correlation-driven network, a random selection of 90% of these pairs is used, and the images are subjected to NSST decomposition. Additionally, a non-repeating subset of 10% of the dataset is extracted to evaluate and test our framework. Note that fine tuning is not applied to the TNO dataset to verify the generalization performance of the method.

The results of image fusion are usually evaluated objectively. However, in most cases, the differences in fusion results obtained by different methods are small, making it difficult to judge using subjective evaluation. Therefore, many metrics for measuring fusion results have been proposed, most of which are designed based on the transfer of edges and information. However, there is no absolute optimal metric, so it is necessary to use multiple metrics to comprehensively evaluate different fusion methods. We use five metrics to quantitatively evaluate different fusion methods, i.e., spatial frequency (SF) [37], entropy (EN) [38], transfer edge information quantity ($Q^{AB/F}$) [39], standard deviation (SD) [40], and average gradient (AG) [41]. For these evaluation metrics, a larger value indicates better fusion performance.

**Parameter initialization.** NSST uses the pyrexc filter for the shearlet transform. We set four levels decomposition of NSST, and the decomposition direction of each level is [8, 8, 8, 16].

For deep learning network training, the batch size is set to 8. We adopt the Adam optimizer with the initial learning rate set to $10^{-3}$. The number of Restormer blocks is 4, with 8 attention heads and 64 dimensions. The dimension of the LT block is same as that of the Restormer. The parameters of the decoder are the same as those of the encoder. And $\alpha_{1}$ to $\alpha_{3}$ are set to 4, 4 and 1.

As other methods do not adjust fusion parameters during experimentation, we ensure the fair comparison of our proposed framework by employing identical parameters in experiments conducted on the two aforementioned datasets.

### 4.2. Comparison with Different Methods

In order to comprehensively evaluate the performance of our method, we compare the proposed ECFuse with twelve other methods on the TNO and LLVIP dataset.

**Figure 7:**
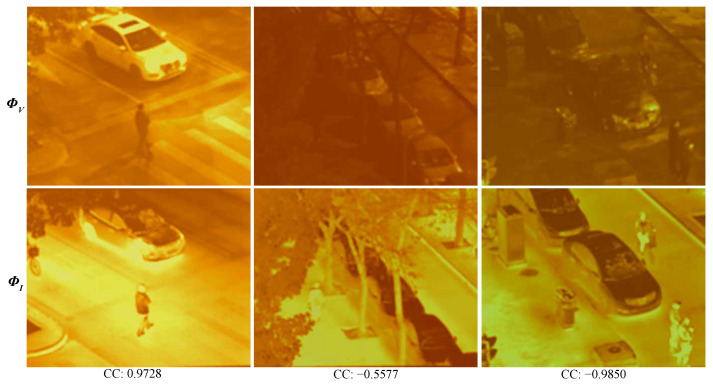
Visualization of the global encoder output.

**Visualization of features.** Figure 7 visualizes the encoder features. Obviously, a larger portion of background information within the feature becomes activated, and these activated regions exhibit relevant characteristics. In this example, the infrared features prominently emphasize object highlights, whereas visible features attentively capture intricate details and textures, thus affirming the successful extraction of modality-specific features. This visualization aligns seamlessly with our prior analysis.

**Figure 8:**
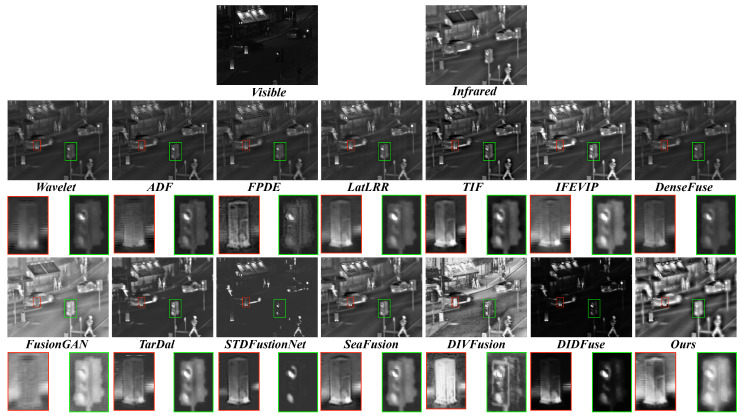
Visual quality comparison of different methods on TNO dataset. For clearer comparison, regions with abundant textures are zoomed in with the red box and green box.

**Figure 9:**
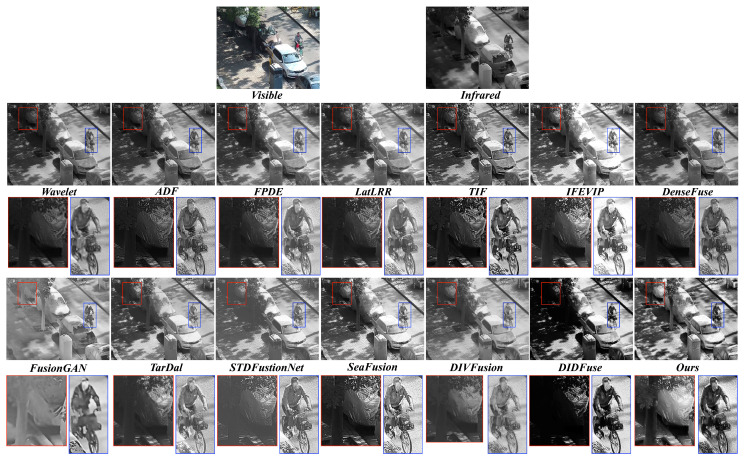
Visual quality comparison of different methods on #260061 image from LLVIP dataset. For clearer comparison, regions with abundant textures are zoomed in with the red box and blue box.

**Qualitative comparison.** We show the qualitative comparison in Figure 8 and Figure 9. Obviously, our method better integrates thermal radiation information in infrared images and detailed texture in visible images.

As shown in Figure 8, our approach is the only one that effectively preserves the object highlighted within the red bounding box and the green box. Our method not only retains the objects within the red box comprehensively but also maintains reasonable contrast and brightness, which proves beneficial for observing objects in dark scenes. Furthermore, concerning the traffic light, wavelet, FPDE, DenseFuse, DIVFusion, STDFusionNet, and DIDFuse do not effectively preserve the integrity of the object. The object’s boundary is closely aligned with the background. Although other methods retain the object’s integrity, their contrast is relatively weak. In contrast, our method’s fused result exhibits a more pronounced contrast between the traffic light and the background. This more salient information is beneficial for accurately discerning object edges and improving our understanding of the scene.

From Figure 9, the car zoomed-in red box is clearly highlighted, even though it is situated in a dark region. This highlighting allows foreground targets to be easily distinguished from the background. Moreover, the background details, which would have been difficult to identify due to low illumination, now possess clear edges and abundant contour information, helping us to better understand the scene. In well-exposed foreground regions, both our method and other approaches retain the main features of the objects. However, FusionGAN and IFEVIP suffer from a significant loss of fine details. While other methods preserve most of the details, our approach excels at enhancing the details of people and other objects with higher contrast. This heightened contrast effectively highlights regions with valuable information, thus improving our focus and understanding of salient targets.

**Quantitative comparison.** Afterward, five metrics are employed to quantitatively compare the above results, which are displayed in Table 1 and Table 2. Our method has excellent performance on most metrics, indicating its suitability for diverse illumination conditions and various target categories. Although the $Q^{AB/F}$ metric does not show significant advantages, the four other metrics demonstrate notable superiority. On the TNO dataset, EN, SF, SD, and AG all exhibit a clear advantage over other methods. On the LLVIP dataset, EN, SF, and SD continue to maintain significant advantages, with AG ranking second but still showing notable superiority compared to other methods.

In summary, the fusion results with high contrast and preserved objects in low-light environments contribute to a better understanding of the scene. The advantages demonstrated in the quantitative evaluation indicate that we retained more information from the source images. Both qualitative and quantitative evaluations show the effectiveness of our method.

### 4.3. Ablation Study

Ablation experiments are set to verify the rationality of the different modules. The same metrics are used to quantitatively validate the fusion effectiveness.

#### 4.3.1. Edge-Consistent Fusion Module Analysis

The role of the edge-consistent fusion module is to adaptively perform region-wise consistency verification fusion using the carefully designed bilateral kernel. The processes of bilateral transpose consistency and smoothing enhances the edges and textures in the activity map adaptively, ensuring the consistency and integrity of textures and edges in the subsequent fusion process, which helps reduce artifacts in the fusion results. The activity-based fusion strategy retains regions with higher information content, which generally correspond to textures and edges. Since this module involves fusion, it cannot be simply removed. To provide a comparison, we select two other common fusion strategies: the pixel-wise average value strategy and the pixel-wise maximum value strategy. Both of these strategies simply fuse each pixel from the source images without considering the integrity of textures and edges.

**Figure 10:**
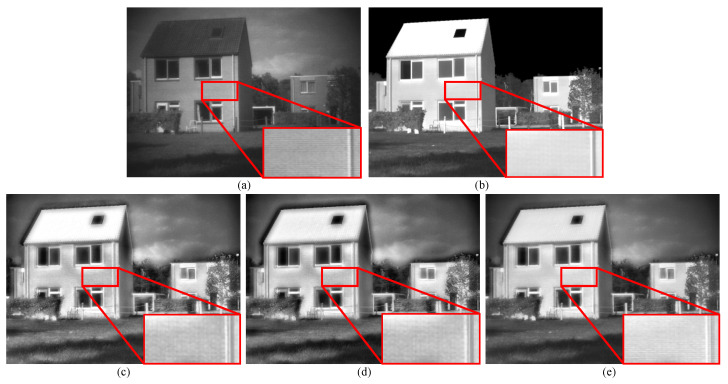
Vision quality comparison of different configurations on TNO dataset. For clearer comparison, regions with abundant textures are zoomed in the red box. (**a**) Visible image. (**b**) Infrared image (**c**) Max fusion scheme. (**d**) Average fusion scheme. (**e**) Ours.

As shown in Figure 10, we replace the proposed edge-consistency fusion module with both the max fusion scheme and average fusion scheme fusion strategies. Observing the drainage pipe region in the zoomed box, it is evident that neither the maximum value nor the mean value strategy manages to preserve sharp and continuous edges. In contrast, our proposed method retains continuous edges and obtains better visual appeal.

From Table 3 and Table 4, we can observe that on both the TNO and LLVIP datasets, our proposed method outperforms the other two methods in most metrics, which aligns with the aforementioned analysis. On the TNO dataset, only $Q^{AB/F}$ falls slightly behind the average fusion scheme, but the difference is minimal. On the LLVIP dataset, only the SD metric lags. In summary, our proposed method outperforms both the average fusion scheme and the maximum fusion scheme.

#### 4.3.2. Correlation-Driven Fusion Network Analysis

The results of the ablation experiments on the correlation-driven deep learning network are presented in Table 5 and Table 6. We simulate multiple comparative scenarios on the correlation loss to validate its effectiveness. Additionally, we compare the performance of using the conventional method alone or the deep learning method alone. Through these comparisons, it is evident that the combination of conventional methods and deep learning methods holds unique advantages.

**Correlation loss.** In Configuration I, we do not use the correlation loss, and the results shows that the correlation loss is necessary for feature decomposition. While there is a slight advantage for one quantitative evaluation metric in both the TNO and LLVIP datasets compared to our method, the difference is minor. On the other hand, our method demonstrates a significant advantage across other metrics. There is no guarantee that the global encoder can learn the global features without correlation loss.

In Configuration II, we modify the loss function $L_{CC}$ to $CC^{2}$, which signifies that the global encoder is intended to extract unique features from different modalities. Subsequent encoders receive cues from these distinctive features while simultaneously learning shared features from both modalities. From the quantitative evaluation results, it is evident that this design brings improvements when compared to excluding the correlation loss. However, there is still a gap when compared to our original proposed loss function. This observation aligns with our earlier analysis, which emphasizes that since infrared and visible images capture the same scene, the background and overarching features, which dominate the image content, are inherently correlated.

In Configuration III, we replace the division operation in the loss function $L_{CC}$ with subtraction, while keeping the objective of the global encoder extracting shared features across modalities unchanged. The quantitative evaluation results indicate that this alternative design yields satisfactory outcomes, achieving optimal results in the SF metric on the TNO dataset and the EN metric on the LLVIP dataset. Nevertheless, taking a comprehensive view, the design involving division remains superior in terms of overall performance. In conclusion, both division and subtraction designs validate the significance of the correlation loss in enhancing the fusion results, thereby affirming the validity of our proposed method.

**Collaboration of the conventional method and the deep learning method.** To validate the effectiveness of integrating deep learning networks with conventional methods, we experiment with Configuration IV and Configuration V. In Configuration IV, only the deep learning network is retained. In this setup, the source images are directly fed into the correlation-driven deep learning network, with the final fused image as the output. Notably, the deep learning network in Configuration IV is trained on the original spatial image dataset, making it suitable for fusing spatial images. In Configuration V, the deep learning network is replaced by a conventional fusion scheme. Table 5 and Table 6 reveal that the fusion method using only deep learning or conventional method has an advantage in the $Q^{AB/F}$ metric, but it significantly lags behind in most other metrics. Thus, from the quantitative evaluation, using only the correlation-driven deep learning network or conventional method is not as effective as our proposed framework.

**Figure 11:**
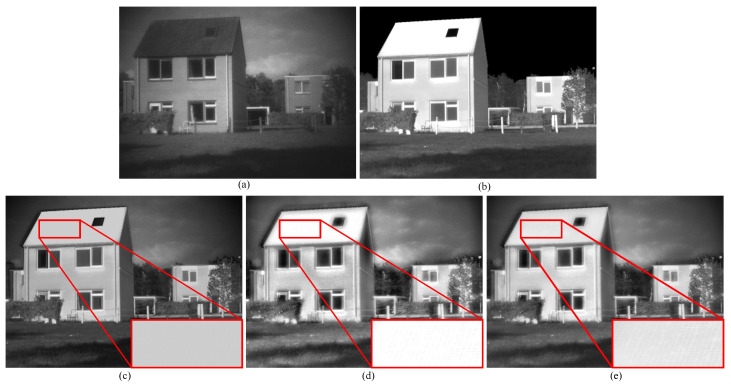
Vision quality comparison of different configurations on TNO dataset. For clearer comparison, regions with abundant textures are zoomed in with the red box. (**a**) Visible image. (**b**) Infrared image (**c**) Only correlation-driven network. (**d**) Only the conventional method. (**e**) Ours.

For a qualitative illustration of the combined effect of deep learning and conventional methods, Figure 11 presents a comparison among the results of using only our proposed deep learning network, only conventional methods, and our proposed framework. As observed in the figure, the fusion outcome from using only deep learning appears generally darker, failing to effectively preserve the features of the thermal targets and lacking in detail richness. The fusion result obtained solely from conventional methods loses the texture of the roof. However, our proposed method not only retains the features of thermal targets but also preserves a greater amount of texture. This demonstrates that the deep learning network we propose, combined with our conventional method-based detail-level fusion strategy, is better suited for complementary utilization.

In summary, the ablation results demonstrate the effectiveness and rationality of our framework design:

### 4.4. Downstream Infrared–Visible Object Detection

**Setup.** The infrared–visible object detection is performed on the $M^{3}\mathrm{FD}$ dataset [5] with 4200 pairs of infrared/visible images, and six categories of labels (i.e., people, car, bus, motorcycle, truck and lamp). It is divided into training/validation/test sets with a proportion of 8:1:1. YOLOv5 [42], a SOTA detector, is employed to evaluate the detection performance with the metric mAP@0.5. The training epoch, batch size, optimizer and initial learning rate are set as 300, 8, SGD optimizer and 1 × 10^−2^, respectively.

**Comparison with SOTA methods.** Table 7 shows that ECFuse has the best detection performance, especially in the motorcycle class, demonstrating that ECFuse can improve the detection accuracy by fusing thermal radiation information.

**Figure 12:**
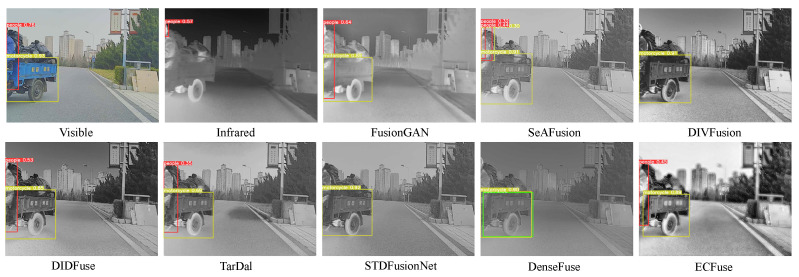
Target detection results on #02788 from $M^{3}\mathrm{FD}$ dataset.

**Figure 13:**
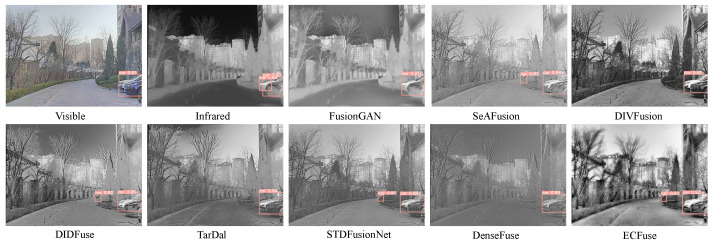
Target detection results on #03738 from $M^{3}\mathrm{FD}$ dataset.

As shown in Figure 12, only the source visible image, FusionGAN, DIDIFuse, TarDal, and our method accurately detect both the human and motorcycle. In Figure 13, the source visible image contains a heavily occluded car that remains undetected in either the isolated visible or infrared images. Among all the methods, SeAFusion, DIDFuse, STDFusionNet, and our method successfully detect this heavily occluded car. In summary, our approach improves the detection accuracy of objects that are challenging to detect in individual modalities.

## 5. Conclusions

In this paper, we propose an edge-consistent and correlation-driven fusion framework for infrared and visible image fusion. Based on NSST decomposition, we obtain the detail layers containing image details and textures, as well as the base layer containing main features. Subsequently, the edge-consistent fusion module adaptively fuses the texture and edges in the detail layers. Then, the correlation-driven deep learning network is proposed to extract the global and modality-specific information in the visible and infrared images and fuse them. Experiments demonstrate that both qualitative and quantitative evaluations are improved. What is more, we also show that ECFuse can boost the performance in downstream infrared–visible object detection.

## Figures and Tables

**Table 1:** Average evaluation metric values of different methods on TNO dataset. The best value in each metric is denoted in **bold**, and the second-best score is highlighted with an underline. ↑ denotes that a higher value indicates a better fusion result.

Methods	EN ↑	SF ↑	SD ↑	$Q^{\mathrm{AB}/F}$↑	AG ↑	Deep Learning
Wavelet [29]	6.3555	0.0250	8.5764	0.2848	2.3745	**✗**
FPDE [33]	6.4162	0.0352	8.6362	0.4810	3.6136	**✗**
ADF [30]	6.4268	0.0364	8.6496	**0.5086**	3.6570	**✗**
LatLRR [32]	6.5153	0.0307	8.6676	0.4169	2.9607	**✗**
TIF [31]	6.6779	0.0411	8.9052	0.4960	3.9615	**✗**
IFEVIP [34]	6.8975	0.0421	9.0887	0.4109	4.1840	**✗**
Densefuse [8]	6.3462	0.0252	8.5718	0.3493	2.5131	**✓**
FusionGAN [6]	6.6434	0.0328	9.1039	0.3810	3.6985	**✓**
TarDal [5]	6.8079	0.0417	9.0444	0.4125	3.8912	**✓**
STDFusionNet [2]	6.9031	0.0455	9.0451	0.4677	4.3846	**✓**
DIDFuse [10]	6.9586	0.0444	9.4718	0.3980	4.2668	**✓**
SeAFusion [7]	7.1337	0.0480	9.5712	0.4872	4.9802	**✓**
DIVFusion [28]	7.5933	0.0528	10.0987	0.3116	5.5602	**✓**
**Ours**	**7.6307**	**0.0576**	**10.1716**	0.3432	**6.0310**	**✓**

**Table 2:** Average evaluation metric values of different methods on LLVIP dataset. The best value in each metric is denoted in **bold**, and the second-best score is highlighted with an underline.

Methods	EN	SF	SD	$Q^{\mathrm{AB}/F}$	AG	Deep Learning
Wavelet [29]	6.8964	0.0241	9.4977	0.1996	2.0167	**✗**
FPDE [33]	6.9161	0.0451	9.4264	0.4909	3.6297	**✗**
ADF [30]	6.9282	0.0489	9.4236	0.5273	3.8861	**✗**
LatLRR [32]	6.9748	0.0450	9.3101	0.4535	3.2089	**✗**
TIF [31]	7.0605	0.0635	9.4683	**0.6354**	4.7440	**✗**
IFEVIP [34]	7.4487	0.0566	9.6836	0.4957	4.1067	**✗**
DenseFuse [8]	6.8899	0.0375	9.4237	0.3530	2.9379	**✓**
FusionGAN [6]	7.0468	0.0293	10.0528	0.2956	2.3374	**✓**
TarDal [5]	7.1872	0.0511	9.6212	0.3857	3.5221	**✓**
STDFusionNet [2]	5.4825	0.0522	6.8897	0.4898	3.4384	**✓**
DIDFuse [10]	6.1477	0.0508	8.0359	0.3605	**6.2487**	**✓**
SeAFusion [7]	7.4457	0.0626	9.8828	0.6254	4.7663	**✓**
DIVFusion [28]	7.5716	0.0547	10.0577	0.3312	4.6006	**✓**
**Ours**	**7.6913**	**0.0667**	**10.1742**	0.4865	5.6376	**✓**

**Table 3:** Bilateral transpose consistency ablation experiment results in the testset of TNO. **Bold** indicates the best value.

	Detail Layers Fusion Scheme	EN	SF	SD	$Q^{\mathrm{AB}/F}$	AG
I	Average	7.6225	0.0484	10.1660	**0.3439**	5.4032
II	Max	7.6276	0.0499	10.1663	0.3320	5.5974
	**Ours**	**7.6307**	**0.0576**	**10.1716**	0.3432	**6.0310**

**Table 4:** Bilateral transpose consistency ablation experiment results in the testset of LLVIP. **Bold** indicates the best value.

	Detail Layers Fusion Scheme	EN	SF	SD	$Q^{\mathrm{AB}/F}$	AG
I	Average	7.6802	0.0482	**10.1943**	0.4577	4.6234
II	Max	7.6844	0.0501	10.1808	0.4268	4.8022
	**Ours**	**7.6913**	**0.0667**	10.1742	**0.4865**	**5.6376**

**Table 5:** Ablation experiment results in the dataset of TNO. The best value in each metric is denoted in **bold**, and the second-best score is highlighted with an underline.

	Configurations	EN	SF	SD	$Q^{\mathrm{AB}/F}$	AG
I	w/o $L_{CC}$	7.5903	**0.0576**	9.8871	0.3356	**6.0326**
II	$L_{CC}\to {\mathrm{CC}}^{2}$	7.6045	**0.0576**	10.0538	0.3386	6.0275
III	$L_{CC}\to \mu -\mathrm{CC}$	7.5893	**0.0576**	10.0852	0.3491	6.0309
IV	Framework → Base layer fusion network	6.9685	0.0423	9.3970	**0.5471**	4.0464
V	Base layer fusion network → Max	7.3181	0.0571	9.7470	0.3805	5.8766
	**Ours**	**7.6307**	**0.0576**	**10.1716**	0.3432	6.0310

**Table 6:** Ablation experiment results in the dataset of LLVIP. The best value in each metric is denoted in **bold**, and the second-best score is highlighted with an underline.

	Configurations	EN	SF	SD	$Q^{\mathrm{AB}/F}$	AG
I	w/o $L_{CC}$	7.5084	0.0662	9.6513	0.4924	5.5903
II	$L_{CC}\to {\mathrm{CC}}^{2}$	7.6911	0.0664	10.2043	0.4870	5.6036
III	$L_{CC}\to \mu -\mathrm{CC}$	**7.6937**	0.0657	10.2064	0.4907	5.5502
IV	Framework → Base layer fusion network	6.9358	0.0497	9.4454	**0.5353**	3.4039
V	Base layer fusion network → Max	7.6775	0.0659	**10.2227**	0.4905	5.5412
	**Ours**	7.6913	**0.0667**	10.1742	0.4865	**5.6376**

**Table 7:** AP0.5(%) values for infrared–visible detection on $M^{3}\mathrm{FD}$ dataset. **Bold** indicates the best value.

	Bus	Car	Lam	Mot	Peo	Tru	mAP@0.5
IR	91.1	85.4	66.5	74.5	76.0	74.1	77.9
VI	91.1	88.6	**78.8**	75.0	67.5	76.4	79.6
FusionGAN	90.7	85.4	66.0	74.5	76.0	77.2	78.3
DIDFuse	93.0	87.8	77.7	69.2	74.4	**81.6**	80.6
STDFusionNet	92.4	87.8	73.0	75.1	73.1	78.2	79.9
TarDal	90.4	87.3	74.1	73.9	75.1	80.5	80.2
SeAFusion	**95.1**	**88.9**	77.6	70.6	**76.2**	77.1	80.9
DenseFuse	94.5	88.7	77.6	73.3	75.0	78.9	81.3
DIVFusion	92.4	87.8	76.9	73.5	72.0	79.4	80.3
**Ours**	92.6	87.2	76.4	**80.2**	74.7	79.7	**81.8**

**Figure A1:**
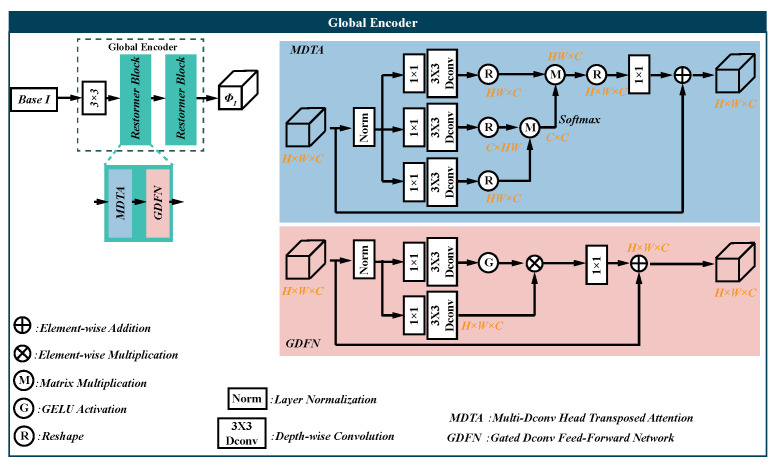
The detail architecture of the global encoder module.

## Data Availability

Source of dataset in experimental analysis: The TNO dataset is obtained from https://figshare.com/articles/dataset/TNO_Image_Fusion_Dataset/1008029, accessed on 20 December 2022. The LLVIP dataset is obtained from https://bupt-ai-cz.github.io/LLVIP, accessed on 25 December 2022. The M${}^{3}$FD dataset is obtained from https://github.com/JinyuanLiu-CV/TarDAL, accessed on 20 May 2023.
